# Activation of nucleus accumbens projections to the ventral tegmental area alters molecular signaling and neurotransmission in the reward system

**DOI:** 10.3389/fnmol.2024.1271654

**Published:** 2024-03-11

**Authors:** Alaa Khayat, Rami Yaka

**Affiliations:** Faculty of Medicine, School of Pharmacy, Institute for Drug Research, The Hebrew University of Jerusalem, Jerusalem, Israel

**Keywords:** cocaine, VTA, NAc, mPFC, optogenetics, GluA1, ERK, inhibitory inputs

## Abstract

The nucleus accumbens (NAc) and the ventral tegmental area (VTA) are integral brain regions involved in reward processing and motivation, including responses to drugs of abuse. Previously, we have demonstrated that activation of NAc-VTA afferents during the acquisition of cocaine conditioned place preference (CPP) reduces the rewarding properties of cocaine and diminished the activity of VTA dopamine neurons. In the current study, we examined the impact of enhancing these inhibitory inputs on molecular changes and neurotransmission associated with cocaine exposure. Our results unveiled significant reductions in extracellular signal-regulated kinase (ERK) levels in the VTA and medial prefrontal cortex (mPFC) of both cocaine-treated groups compared with the saline control group. Furthermore, optic stimulation of NAc-VTA inputs during cocaine exposure decreased the expression of GluA1 subunit of AMPA receptor in the VTA and mPFC. Notably, in the NAc, cocaine exposure paired with optic stimulation increased ERK levels and reduced GluA1 phosphorylation at Ser845 as compared with all other groups. Additionally, both cocaine-treated groups exhibited decreased levels of GluA1 phosphorylation at Ser831 in the NAc compared with the saline control group. Moreover, cocaine exposure led to reduced ERK, GluA1, and GluA1 phosphorylation at Ser845 and Ser831 in the mPFC. Augmentation of GABAergic tone from the NAc during cocaine conditioning mitigated changes in GluA1 phosphorylation at Ser845 in the mPFC but reduced ERK, GluA1, and GluA1 phosphorylation at Ser831 compared with the saline control group. Interestingly, enhancing GABAergic tone during saline conditioning decreased GluA1 phosphorylation at Ser831 compared with the saline control group in the mPFC. Our findings highlight the influence of modulating inhibitory inputs from the NAc to the VTA on molecular signaling and glutamatergic neurotransmission in cocaine-exposed animals. Activation of these inhibitory inputs during cocaine conditioning induced alterations in key signaling molecules and AMPA receptor, providing valuable insights into the neurobiological mechanisms underlying cocaine reward and cocaine use disorder. Further exploration of these pathways may offer potential therapeutic targets for the treatment of substance use disorder.

## Introduction

Substance use disorder (SUD) is a chronic brain disease characterized by compulsive drug-seeking and drug-taking behaviors, regardless of the negative consequences (Leshner, 2022). One of the prominent challenges in SUD is the enduring susceptibility to relapse, often triggered by drug-related cues (Kalivas and Volkow, 2005; Pickens et al., 2011; Perry et al., 2014), indicating long-lasting alterations in the brain. Despite extensive research efforts dedicated to unraveling the neuropharmacological mechanisms underlying SUD, effective treatment options have yet to be discovered.

The brain’s reward system is a complex network of interconnected regions that play a significant role in motivation, reward processing, and SUD (Beart et al., 1979; Johnson and North, 1992). The key component of the reward system is the ventral tegmental area (VTA), which primary contains dopaminergic cells that release dopamine in the nucleus accumbens (NAc) and PFC (Feltenstein and See, 2008). In addition to dopamine cells, the VTA also contains GABAergic neurons that modulate the activity of dopaminergic neurons (Johnson and North, 1992). The VTA is the main source of dopamine in the reward system, making its modulation of great physiological and pathophysiological importance. As a result, inhibitory GABAergic synapses constitute the predominant type of synapse onto midbrain DA neurons, comprising 50–80% of all synapses (Bayer and Pickel, 1991; Johnson and North, 1992; Cruz et al., 2004; Henny et al., 2012; Edwards et al., 2017). The inhibitory effects of GABA are mediated by both fast ionotropic GABA_A_ receptors (GABA_A_Rs) and slow metabotropic GABA_B_ receptors (GABA_B_Rs). GABA_A_Rs are ligand-gated ion channels that mediate fast synaptic inhibition by allowing the flow of chloride ions into the cell, which hyperpolarizes the membrane potential and reduces the excitability of the neuron. GABA_A_Rs are widely distributed in the brain and are involved in the rapid control of neuronal excitability. GABA_B_Rs, on the other hand, are G protein-coupled receptors that mediate slow synaptic inhibition through the activation of intracellular signaling pathways. They are located primarily at presynaptic terminals and are involved in the modulation of neurotransmitter release. The activation of GABA_B_Rs leads to the opening of potassium channels, which hyperpolarizes the membrane potential and reduces the excitability of the neuron.

There are several sources of GABAergic inputs to the VTA, including the Rostromedial Tegmental Nucleus (RMTg) (Jhou et al., 2009; Hong et al., 2011; Lammel et al., 2012), the ventral pallidum (Geisler and Zahm, 2005), and the GABAergic neurons within the VTA itself (Steffensen et al., 1998; Tan et al., 2012; Van Zessen et al., 2012). Additionally, the NAc, which is a major target of VTA dopamine (DA) neurons, sends GABAergic projections back to the VTA to regulate their activity (Xia et al., 2011; Polter et al., 2018). The NAc plays a major role in reward processing and provides motivation in response to both natural rewards and drugs of abuse (Feltenstein and See, 2008). In this study, we investigate the role of NAc inhibitory inputs to the VTA. In the NAc, there are two main subpopulations of MSNs based on the DA receptors they express: D1 receptor-expressing MSNs and D2 receptor-expressing MSNs. D1R-expressing MSNs, also known as direct pathway neurons, project directly to the VTA and extend their projections to the ventral pallidum (VP). Conversely, D2R-expressing MSNs, also known as indirect pathway neurons, project specifically to the ventral pallidum (VP). This indirect pathway involves GABAergic projections from the VP to the VTA, ultimately resulting in the inhibition of DA neuron activity (Lu et al., 1998; Gagnon et al., 2017). The precise characteristics of the NAc projections to the VTA and their role in regulating DA neuron activity are still subjects of active research and debate. Some studies have suggested that the NAc sends direct GABAergic projections to the VTA, which can inhibit DA neuron activity (Edwards et al., 2017; Yang et al., 2018), while other studies propose that the influence of the NAc on the VTA is mediated indirectly through VTA GABAergic interneurons, resulting in the disinhibition of DA neurons (Xia et al., 2011). Further investigation is required to fully comprehend the complexity of this circuit and its role in regulating reward processing and a motivated behavior.

In our previous research, we provided compelling evidence for the involvement of inhibitory inputs from the NAc to the VTA in mitigating the rewarding properties of cocaine and reducing the activity of VTA dopaminergic neurons (Weitz et al., 2021). The present study aims to deepen our understanding of the underlying neurobiological mechanisms that regulate cocaine reward and the associated neuronal adaptations. Specifically, we focused on examining the alteration in the expression and function of the extracellular signal-regulated kinase (ERK) (Berhow et al., 1996; Valjent et al., 2000, 2004; Mattson et al., 2005; Qin et al., 2005), which was implicated in synaptic plasticity. The phosphorylation state of ERK, indicating its activation, is frequently evaluated to assess its participation in signaling pathways and its subsequent involvement in cellular events (Bingor et al., 2021). The GluA1 subunit of the AMPA receptor (Borgland et al., 2004; Mameli et al., 2009; Lüscher and Malenka, 2011) is a key player in learning and memory processes. Phosphorylation of specific serine residues on the intracellular tail of GluA1, such as S831 and S845, is known to be critically involved in the trafficking and function of GluA1 and can influence its localization to the synapse and its overall synaptic efficacy (Schumann and Yaka, 2009; Bingor et al., 2020). Our research focused on how activating the projections from the NAc to the VTA impacts these molecules within the reward-related regions of the brain—the VTA, NAc, and medial prefrontal cortex (mPFC). Previous studies have demonstrated that dysfunction of the mPFC can contribute to addictive behaviors and substance use disorder (SUD). The inability of individuals to control their impulses and make rational decisions in the face of addictive drugs can, in part, be attributed to impaired prefrontal cortical function (Jentsch and Taylor, 1999; Goldstein and Volkow, 2011). Therefore, we also examined whether activation of the NAc-VTA projections will be reflected in molecular adaptations in the mPFC.

To probe these mechanisms, we employed a powerful combination of optogenetics and the repeated exposure place preference (RePP) paradigm. Optogenetics allows for precise and selective activation of specific neural circuits, enabling us to elucidate the effects of activating the NAc-VTA projections on the targeted brain regions. The RePP paradigm, a modified version of the conditioned place preference (CPP) paradigm, offers a more efficient assessment of behavioral outcomes, resulting from NAc-VTA projection activation in response to cocaine exposure.

Our study aims to uncover the neural mechanisms of cocaine reward and cocaine use disorder by investigating the consequences of activating NAc-VTA projections. By utilizing optogenetics and the RePP paradigm, we seek to shed light on the behavioral and molecular implications within critical brain regions, leading to potential therapeutic targets for combating SUD. This research represents a step forward in understanding the molecular mechanism that underlie a drug reward.

## Materials and methods

### Animals

Sprague–Dawley (SD) male rats (Envigo), aged approximately 4 weeks old, weighing between 50 and 70 g were group-housed in the animal facility under specific conditions. The ambient temperature was maintained at 22°C, and the rats were kept on a 12-h light/dark cycle, with the lights turned on at 7:00 a.m. The animals had access to food and water *ad libitum*, allowing them to consume as much as they desired. During the behavioral studies, the experiments were conducted in a dark room with only red light present. This choice of light is undetectable by rodents. All procedures performed in this study were approved by the Institutional Animal Care Committee (IACUC; #1165684) of the Hebrew University in Jerusalem, Israel. Furthermore, the experimental design and execution were conducted in a manner that prioritized the welfare and minimized any potential discomfort experienced by the animals.

### Stereotactic surgery

On the day of surgery, the rats were anesthetized using a combination of xylazine and ketamine (in a ratio of 0.15:0.85). A microinjection needle with a diameter of 33 gauge was connected to a 10-μl syringe (Hamilton, United States). The needle was inserted unilaterally into the NAc (coordinates: AP: +1.75, ML: 1.48, DV: −6.2). A purified adeno-associated virus (AAV) (2 × 10^9^ units; 1 μL volume) encoding hChR2 (H134R)-m-Cherry under the control of the human synapsin 1 gene promoter was injected for 5 min, with the syringe left in place for an additional 7 min. The injections were performed using a Digital Lab Standard™ Stereotaxic instrument (Stoelting Co. IL, United States). Following the surgery, the rats were housed individually in separate cages for 2 weeks to allow for recovery and complete expression of the viral vector. After 2 weeks of injecting the channel rhodopsin-containing virus into the NAc, the animals underwent another surgery in which an optic fiber was implanted above the VTA (coordinates: AP: -5.1, ML: 0.7, DV: −8.1). The rats were given 1 week to recover before the initiation of the behavioral experiments.

### Conditioning apparatus

The CPP apparatus used in this study was obtained from Med Associates, USA. It consists of a three-compartment elongated rectangular apparatus. The middle compartment serves as a neutral zone, measuring 12 cm x 21 cm, and it separates the two elongated compartments. One of the compartments is characterized by white-colored walls and a wire mesh flooring measuring 28 cm x 21 cm. The other compartment features black-colored walls and a steel rod flooring of the same dimensions. To track the time spent in each compartment, infrared photobeam crossings were positioned at the bottom of the walls. These photobeams detect the movement of the animals and record the time spent in each compartment during the CPP experiments.

### Repeated-exposure place preference (RePP)

After 3 weeks of injection of the channel rhodopsin-containing virus to the NAc, rats underwent a habituation session in the CPP apparatus. The least preferred compartment for each subject was then designated as the drug-paired compartment (biased). Following habituation session, rats underwent six conditioning sessions for 3 consecutive days, with two sessions per day. They were randomly assigned to one of the four groups: (1) saline control group: These rats received saline (1 mL/kg, i.p.) in both sessions without any optic stimulation; (2) saline control group with optic stimulation: These rats received saline (1 mL/kg, i.p.) in both sessions, with optic stimulation only during the morning sessions; (3) cocaine control group: These rats received cocaine (15 mg/kg, i.p.) in the morning session and saline (1 mL/kg, i.p.) in the afternoon session, without any optic stimulation; and (4) cocaine-treated group: These rats received cocaine (15 mg/kg, i.p.) in the morning session and saline (1 mL/kg, i.p.) in the afternoon session, with optic stimulation only during the cocaine sessions. In all cases, an optic cannula was connected to the light source, and optic stimulation was administered using an ultra high-power LED (Prizmatix, Israel). For NAc stimulation, the optic stimulation protocol involved delivering stimulation at 20 Hz for 10 s per minute (Larson et al., 2015). On the 5th day of the experiment, a CPP test was conducted to assess the CPP. The CPP score was calculated as a percentage using the following formula: CPP score = 100 * [(time spent in the drug/saline-paired compartment) - (time spent in the saline-paired compartment)] / [(time spent in the drug/saline-paired compartment) + (time spent in the saline-paired compartment)]. This CPP score measurement provided an assessment of the CPP, indicating the relative preference for the drug-paired compartment compared with the saline-paired compartment.

### Western blotting analysis

Rats were first anesthetized using isoflurane. Following anesthesia, they were decapitated, and their brains were immediately removed. Coronal sections, with each being approximately 1 mm thick, containing the VTA, NAc, and mPFC were obtained, and these sections were then microdissected bilaterally on an ice-cold platform and promptly transferred to liquid nitrogen to preserve the tissue. The microdissected tissue samples were homogenized using a homogenization buffer consisting of 320 mM sucrose, 10 mM Tris–HCl (pH 7.4), 1 mM EDTA, 1 mM EGTA, a protease inhibitor cocktail (Sigma, P8340), and phosphatase inhibitors (1 mM Na_3_VO_4_ and 5 mM NaF by Sigma-Merck, Darmstadt, Germany). The purpose of the homogenization step is to break down the tissue and release the proteins of interest. To determine the total protein concentration in the brain homogenates, the Pierce™ BCA Protein Assay Kit (Pierce, IL, United States) was utilized, with bovine serum albumin (BSA) as a standard. The protein samples were then boiled for 5 min at 95°C to denature the proteins and loaded (20 μg protein/lane) onto 8–12.5% SDS-PAGE gels. Electrophoresis was performed to separate the proteins based on their molecular weight, and the separated proteins were transferred onto a nitrocellulose blotting membrane (TAMAR, Germany). The purpose of this transfer was to immobilize the proteins on the membrane for subsequent antibody detection. Following the transfer, the membranes were incubated at room temperature for 1 h in a blocking buffer containing 5% non-fat dry milk. This step helps prevent non-specific binding of antibodies. The membranes were then incubated overnight at 4°C with the desired primary antibodies. The primary antibodies used in this study were Phospho ERK (Thr202/Tyr204) (cat. no. 91015; 1:1,500; Cell Signaling), ERK (cat. no. 9102 s; 1:1000; Cell Signaling), GluR1 (cat. no. AB1504; 1:2,000; Merck), Anti-phospho-GluR1 (Ser845) (cat. no. AB 5849; 1:500; Merck), Anti-phospho-GluR1 (Ser831) (cat. no. 04–823; 1:500; Merck), and Beta Actin (cat. no. ab8227; 1:7,000; Abcam). After primary antibody incubation, the membranes were washed to remove unbound antibodies and then incubated with the appropriate HRP-conjugated secondary antibody for 1.5 h at room temperature. The secondary antibody recognizes and binds to the primary antibody, allowing for the detection of the target proteins. The blots on the membrane were detected and quantified using the Clarity™ Western ECL System and the BioRad ChemiDoc™ XRS+ Imaging System (BioRad, CA, United States). This detection method utilizes the chemiluminescence test to visualize the antibodies bound to the membrane and quantify the protein bands.

### Statistical analysis

For the behavioral and biochemical analyses, the effect of treatment was assessed using a one-tailed t-test and a two-tailed Student’s unpaired t-test for pairwise comparisons. These tests were chosen based on the specific hypotheses and research questions being investigated. To analyze multiple comparisons between groups, a two-way analysis of variance (ANOVA) was employed. In cases where significant differences were observed in the two-way ANOVA, post-hoc Tukey honestly significant difference (HSD) tests were conducted. This approach allowed us to thoroughly examine the impact of the treatment and identify any significant variations between the experimental groups. Data are presented as mea*n* ± SEM (standard error of the mean). The accepted level of significance for all tests was set at a value of *p* of <0.05, which is denoted by asterisks in the figures. The figure legends provide information regarding the group sizes and significant differences observed. Statistical analysis was performed using Prism 8 software.

## Results

### Optic stimulation of NAc afferents to the VTA during the acquisition phase of RePP significantly reduced cocaine CPP

**Figure 1:**
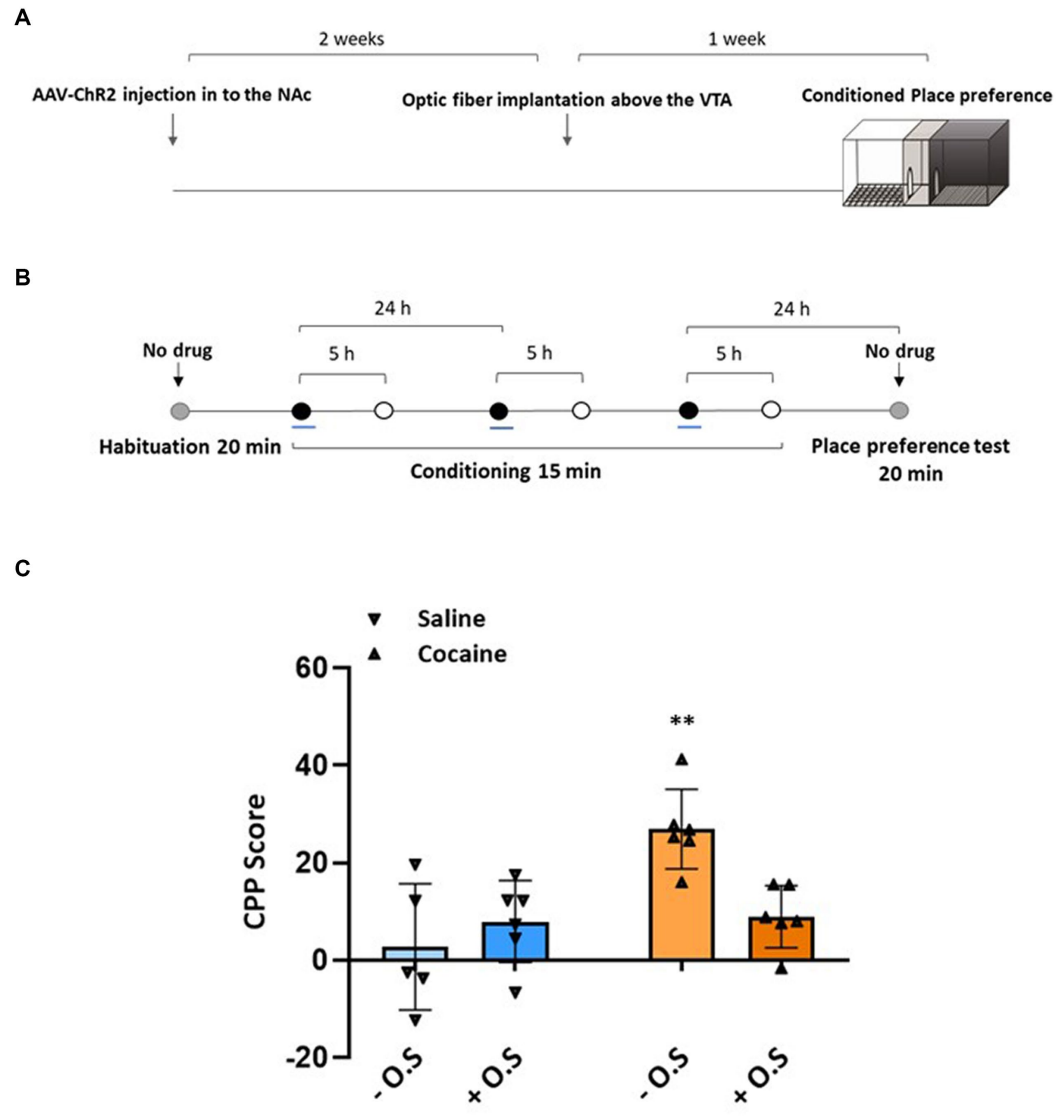
Activation of NAc afferents during cocaine exposure reduced the expression of CPP. **(A)** An illustration demonstrating AAV-ChR2 injection in the NAc and optic fiber implantation above the VTA. This set-up allows for the optic stimulation of VTA-innervating afferents during RePP. **(B)** For the CPP study, rats were randomly assigned to four groups. Following habituation (Hab.), the first control group received an injection of saline (1 mL/kg) without optic stimulation. The second control group received an injection of saline (1 mL/kg) and underwent optic stimulation. The third control group received an injection of cocaine (15 mg/kg) without optic stimulation. Finally, the fourth group received an injection of cocaine (15 mg/kg) and underwent optic stimulation during conditioning. Following 1 day of the termination of conditioning (day 5, test), the rats were tested for their preference to the cocaine-paired compartment. (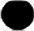
= Rats confined to black or white compartment after injection of saline (1 mL/kg) or cocaine (15 mg/kg) with or without optic stimulation, 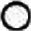
= Rats confined to black or white compartment after injection of saline (1 mL/kg), 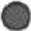 = Free exploration). C. Graph depicting preference values expressed as the mean CPP score ± SEM in NAc-injected animals (*n* = 5, 6, 6, and 6 rats for the saline -, saline +, cocaine −, and cocaine + O.S. groups, respectively) on the test day. **Cocaine − O.S. group differs significantly from saline - O.S. and saline + O.S. groups (*p* < 0.01). *The cocaine − O.S. group differs significantly from the cocaine + O.S. group (*p* < 0.05) based on a two-way ANOVA test [F_OPTIC STIMULATION_ (1,19) = 2.842, *p* > 0.05, F_GROUP_ (1,19) = 11.02, *p* < 0.01, F_INTERACTION_ (1,19) = 9.370, *p* < 0.01]. Tukey’s multiple comparison test was used for post-hoc analysis. (− O.S refers to - optic stimulation, whereas + O.S refers to + optic stimulation).

Previously, we demonstrated that the exogenous activation of NAc afferents in the VTA during the acquisition phase of cocaine conditioning led to the elimination of animals’ preference for the cocaine-associated chamber and a reduction in VTA neuronal activity (Weitz et al., 2021). To gain further insights into the effects of this activation on cocaine-induced molecular changes in reward-related brain areas, including the VTA, NAc, and mPFC, and to determine if the activation itself causes molecular changes, we conducted a shortened CPP experiment with four different groups of animals. The first group received a saline injection (1 mL/kg) without optic stimulation, serving as the saline control group. The second group received a saline injection (1 mL/kg) and underwent optic stimulation. The third group received an injection of cocaine (15 mg/kg) without optic stimulation. Finally, the fourth group received an injection of cocaine (15 mg/kg) and underwent optic stimulation during the conditioning phase (Figures 1A,B).

Consistent with the findings of the previous longer CPP experiment, the RePP experiment yielded similar results. As expected, all animals that received cocaine without optic stimulation displayed a significant preference for the cocaine-associated chamber [one-sample *t* test: *t* (5) = 8.062, *p* = 0.0005]. However, when NAc afferents were stimulated during cocaine conditioning, the animals exhibited a significant reduction in preference compared with the cocaine without the optic stimulation group (unpaired t-test: t(10) = 4.257, *p* < 0.01). Importantly, optic stimulation of the NAc in saline-injected animals did not have an impact on their preference [unpaired *t*-test: *t* (9) = 0.8072, *p* > 0.05]. Post-hoc Tukey HSD tests confirmed that the group receiving cocaine without optic stimulation differs significantly from the saline control group (*p* < 0.01), the Cocaine + optic stimulation group (*p* < 0.05), and a two-way ANOVA test (Figure 1C).

The findings of this study provide further evidence for the role of NAc afferents in modulating the rewarding properties of cocaine. The observed reduction in preference for the cocaine-associated chamber in animals undergoing NAc activation during cocaine conditioning suggests that the activation of NAc afferents interferes with the rewarding effects of cocaine. It is important to note that this specificity of these effects to cocaine-induced reward has not been tested with other rewarding stimuli, such as food or sex. Therefore, it remains unclear whether NAc afferent activation has a similar impact on natural rewards. Furthermore, the lack of impact on preference in saline-injected animals receiving NAc optic stimulation highlights that optic stimulation itself does not cause aversion.

### Enhanced GABAergic tone from the NAc during conditioning to cocaine alters molecular signaling in the VTA, NAc, and mPFC

In our previous research, we demonstrated that activating inhibitory inputs from the RMTg to the VTA during the cocaine conditioning period reduced cocaine-induced molecular changes in the VTA, NAc, and mPFC. We, therefore, hypothesized that activating inhibitory inputs from the NAc to the VTA would yield similar molecular changes. To test this hypothesis, we sacrificed the animals 45 min after the RePP test, and the VTA, NAc, and mPFC regions were collected for subsequent Western blotting (WB) analysis. We specifically investigated changes in the AMPA receptor GluA1 subunit and ERK signaling.

**Figure 2:**
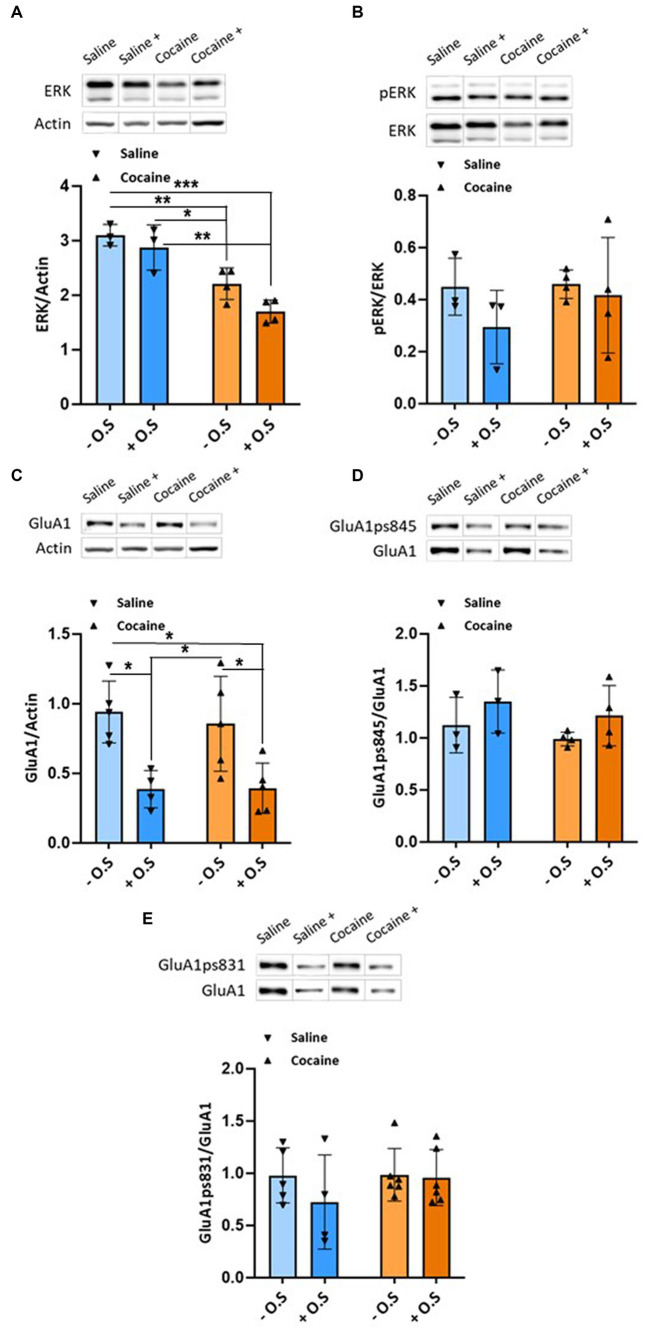
Enhanced GABAergic tone from the NAc during conditioning to saline or cocaine decreases GluA1 levels in the VTA. All rats were euthanized 45 min after the RePP test. Then, their VTA were taken out and homogenized. Samples were resolved by SDS-PAGE, and WB membranes were probed with the appropriate antibodies. **(A)** The bar histograms depict the level of ERK divided by Actin, and rats treated with cocaine (15 mg/kg) without optic stimulation, and rats treated with cocaine with optic stimulation displayed significantly lower values compared with the saline groups (*p* < 0.05), as determined by a two-way ANOVA test [F_OPTIC STIMULATION_ (1,10) = ***5.746***, *p* < 0.05, F_GROUP_ (1,10) = 45.24, *p* < 0.0001, F_INTERACTION_ (1,10) = 0.8606, *p* > 0.05, followed by Tukey’s multiple comparison test]. (*n* = 3 for the saline groups, *n* = 4 for the cocaine groups). **(B)** The bar histograms depict the level of pERK (Thr202/Tyr204) divided by ERK antibodies; no significant difference was found (*p* > 0.05), as determined by a two-way ANOVA test [F_OPTIC STIMULATION_ (1,10) = 1.515, *p* > 0.05, F_GROUP_ (1,10) = 0.6789, *p* > 0.05, F_INTERACTION_ (1,10) = 0.4926, *p* > 0.05, followed by Tukey’s multiple comparison test]. (*n* = 3 for the saline groups, *n* = 4 for the cocaine groups). **(C)** The bar histograms depict the level of GluA1 divided by Actin, and rats treated with saline or cocaine with optic stimulation displayed significantly lower values compared with rats treated with saline or cocaine without optic stimulation (*p* < 0.05), as determined by a two-way ANOVA test [F_OPTIC STIMULATION_ (1,15) = 21.58, *p* < 0.001, F_GROUP_ (1,15) = 0.1269, *p* > 0.05, F_INTERACTION_ (1,15) = 0.1785, *p* > 0.05, followed by Tukey’s multiple comparison test]. (*n* = 5, 4, 5, and 5 for saline -, saline +, cocaine-, and cocaine+ O.S. groups, respectively). **(D)** The bar histograms depict the level of GluA1ps845 divided by GluA1 antibodies, and no significant difference was found, as determined by a two-way ANOVA test [F_OPTIC STIMULATION_ (1,10) = 2.936, *p* > 0.05, F_GROUP_ (1,10) = 1.063, *p* > 0.05, F_INTERACTION_ (1,10) = 0.00007179, *p* > 0.05, followed by Tukey’s multiple comparison test]. (*n* = 3 for the saline groups, *n* = 4 for the cocaine groups). **(E)** The bar histograms depict the level of GluA1ps831 divided by GluA1 antibodies, and no significant difference was found, as determined by a two-way ANOVA test [F_OPTIC STIMULATION_ (1,17) = 1.111, *p* > 0.05, F_GROUP_ (1,17) = 0.8050, *p* > 0.05, F_INTERACTION_ (1,17) = 0.7182, *p* > 0.05, followed by Tukey’s multiple comparison test]. (*n* = 5, 4, 6, and 6 for saline -, saline +, cocaine-, and cocaine+ O.S. groups, respectively). (− O.S refers to - optic stimulation, whereas + O.S refers to + optic stimulation).

In the VTA, our results revealed that rats treated with cocaine, with or without optic stimulation, display significantly lower levels of ERK compared with the saline control groups (*p* < 0.05), as determined by a two-way ANOVA test [F_OPTIC STIMULATION_ (1,10) = 5.746, *p* < 0.05, F_GROUP_ (1,10) = 45.24, *p* < 0.0001, F_INTERACTION_ (1,10) = 0.8606, *p* > 0.05], followed by Tukey’s multiple comparison test (Figure 2A), without affecting the levels of phopsphorylated ERK (Figure 2B). Furthermore, rats treated with saline or cocaine with optic stimulation displayed significantly lower levels of GluA1 compared with rats treated with saline or cocaine without optic stimulation (*p* < 0.05), as determined by a two-way ANOVA test [F_OPTIC STIMULATION_ (1,15) = 21.58, *p* < 0.001, F_GROUP_ (1,15) = 0.1269, *p* > 0.05, F_INTERACTION_ (1,15) = 0.1785, *p* > 0.05], followed by Tukey’s multiple comparison test (Figure 2C).

**Figure 3:**
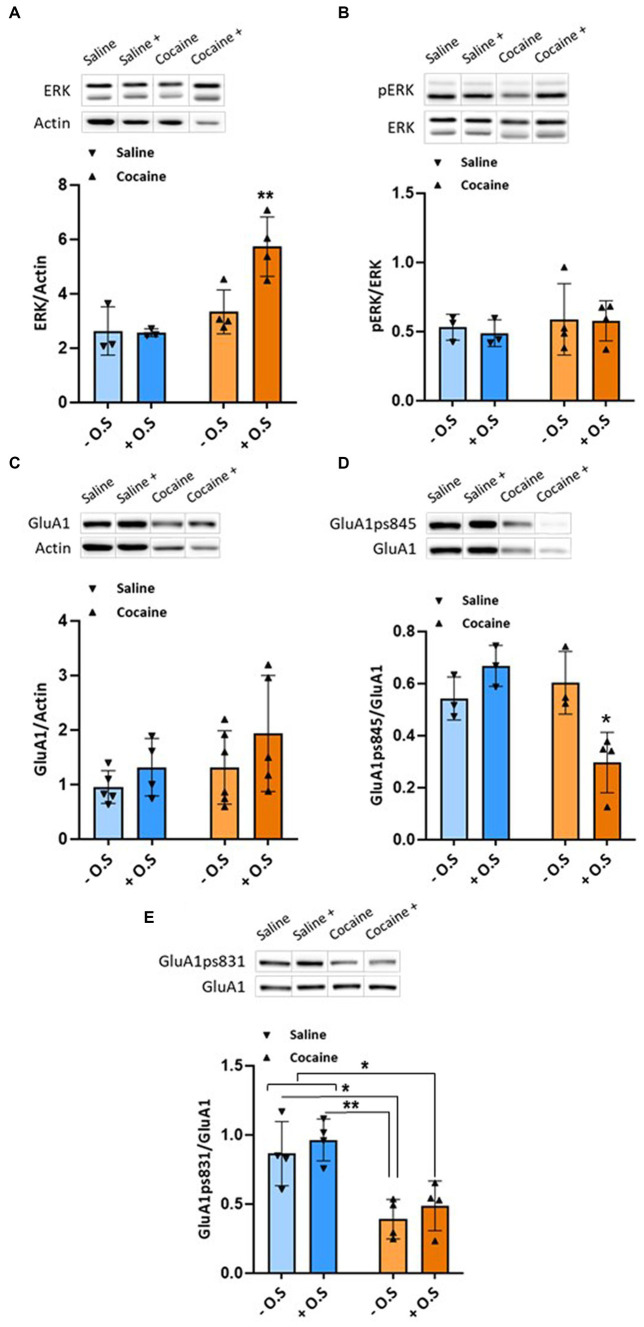
Enhanced GABAergic tone from the NAc during conditioning to cocaine significantly increases ERK level and decreases GluA1ps845 and GluA1ps831 levels in the NAc. All rats were euthanized 45 min after the RePP test. Then, their NAc were taken out and homogenized. Samples were resolved by SDS-PAGE, and WB membranes were probed with the appropriate antibodies. **(A)** The bar histograms depict the level of ERK divided by Actin, rats treated with cocaine (15 mg/kg) with optic stimulation displayed significantly higher values compared with all other groups (*p* < 0.05), as determined by a two-way ANOVA test [F_OPTIC STIMULATION_ (1,10) = 6.563, *p* < 0.05, F_GROUP_ (1,10) = 17.97, *p* < 0.01, F_INTERACTION_ (1,10) = 7.29, *p* < 0.05, followed by Tukey’s multiple comparison test]. (*n* = 3 for the saline groups, *n* = 4 for the cocaine groups). **(B)** The bar histograms depict the level of pERK (Thr202/Tyr204) divided by ERK antibodies; no significant difference was found (*p* > 0.05), as determined by a two-way ANOVA test [F_OPTIC STIMULATION_ (1,10) = 0.083, *p* > 0.05, F_GROUP_ (1,10) = 0.6084, *p* > 0.05, F_INTERACTION_ (1,10) = 0.0061, *p* > 0.05, followed by Tukey’s multiple comparison test]. (*n* = 3 for the saline groups, *n* = 4 for the cocaine groups). **(C)**. The bar histograms depict the level of GluA1 divided by Actin, and no significant difference was found (*p* > 0.05), as determined by a two-way ANOVA test [F_OPTIC STIMULATION_ (1,16) = 2.382, *p* > 0.05, F_GROUP_ (1,16) = 2.350, *p* > 0.05, F_INTERACTION_ (1,16) = 0.1627, *p* > 0.05, followed by Tukey’s multiple comparison test]. (*n* = 5, 4, 6, and 5 for saline -, saline +, cocaine-, and cocaine+ O.S. groups, respectively). **(D)** The bar histograms depict the level of GluA1ps845 divided by GluA1 antibodies, and rats treated with cocaine (15 mg/kg) with optic stimulation displayed significantly lower values compared with all other groups (*p* < 0.05), as determined by a two-way ANOVA test [F_OPTIC STIMULATION_ (1,9) = 2.472, *p* > 0.05, F_GROUP_ (1,9) = 7.298, *p* < 0.05, F_INTERACTION_ (1,9) = 14.07, *p* < 0.01, followed by Tukey’s multiple comparison test]. (*n* = 3 for saline -, saline +, and cocaine - O.S. groups, *n* = 4 for cocaine + O.S. group, respectively). **(E)** The bar histograms depict the level of GluA1ps831 divided by GluA1 antibodies, and rats treated with cocaine (15 mg/kg) without optic stimulation and rats treated with cocaine with optic stimulation displayed significantly lower values compared with the saline groups (*p* < 0.05), as determined by a two-way ANOVA test [F_OPTIC STIMULATION_ (1,12) = 1.185, *p* > 0.05, F_GROUP_ (1,12) = 27.96, *p* < 0.001, F_INTERACTION_ (1,12) = 0.0002625, *p* > 0.05, followed by Tukey’s multiple comparison test]. (*n* = 4 for all groups). (− O.S refers to - optic stimulation, whereas + O.S refers to + optic stimulation).

In the NAc, no change in GluA1 levels was detected (Figure 3C), However, we observed decreased levels of GluA1 phosphorylation at Ser831 in both cocaine groups compared with the saline control groups (*p* < 0.05), as determined by a two-way ANOVA test [F_OPTIC STIMULATION_ (1,12) = 1.185, *p* > 0.05, F_GROUP_ (1,12) = 27.96, *p* < 0.001, F_INTERACTION_ (1,12) = 0.0002625, *p* > 0.05], followed by Tukey’s multiple comparison test (Figure 3E). Optical stimulation of NAc-VTA inputs in rats of both cocaine groups significantly increased ERK levels compared with all other groups (*p* < 0.05), as determined by a two-way ANOVA test [F_OPTIC STIMULATION_ (1,10) = 6.563, *p* < 0.05, F_GROUP_ (1,10) = 17.97, *p* < 0.01, F_INTERACTION_ (1,10) = 7.29, *p* < 0.05], followed by Tukey’s multiple comparison test (Figure 3A), while no change in phosphorylated ERK was detected (Figure 3B). In addition, it decreased GluA1 phosphorylation at Ser845 in the cocaine-treated group compared with all other groups (*p* < 0.05), as determined by a two-way ANOVA test [F_OPTIC STIMULATION_ (1,9) = 2.472, *p* > 0.05, F_GROUP_ (1,9) = 7.298, *p* < 0.05, F_INTERACTION_ (1,9) = 14.07, *p* < 0.01], followed by Tukey’s multiple comparison test (Figure 3D).

**Figure 4:**
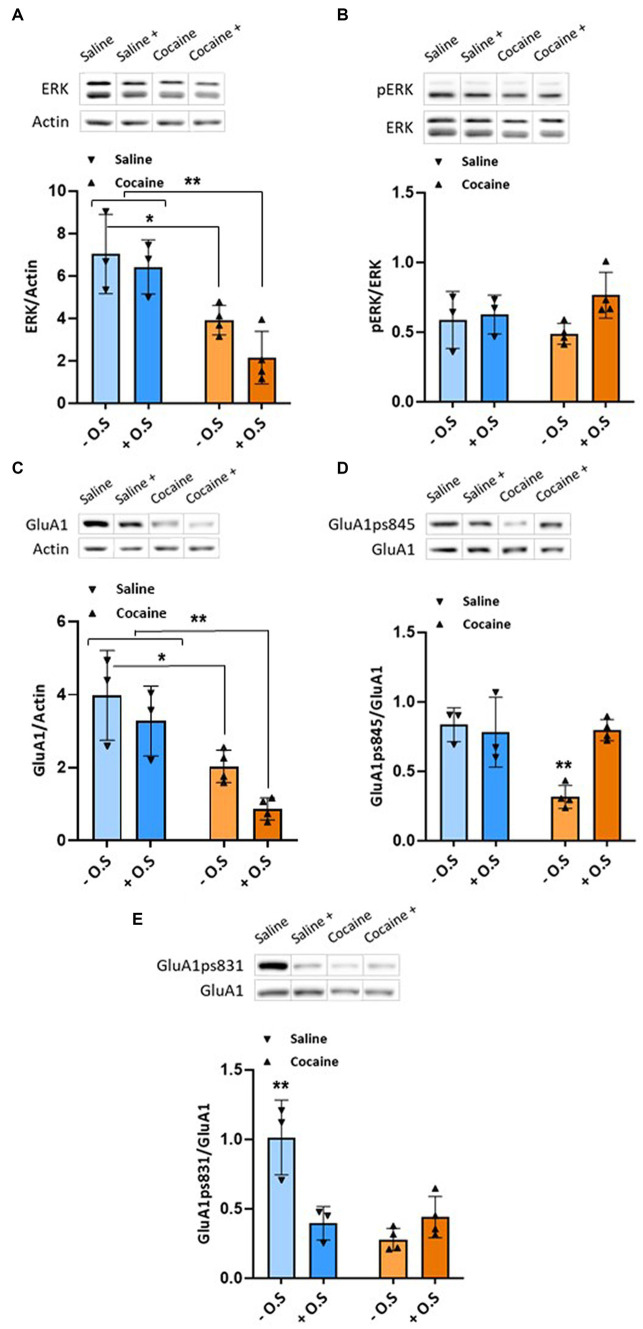
Enhanced GABAergic tone from the NAc during conditioning to cocaine significantly decreases ERK, GluA1, and GluA1ps831 levels, whereas it attenuates changes in GluA1ps845 in the mPFC. All rats were euthanized 45 min after the RePP test. Then, their mPFC were taken out and homogenized. Samples were resolved by SDS-PAGE, and WB membranes were probed with the appropriate antibodies. **(A)** The bar histograms depict the level of ERK divided by Actin, rats treated with cocaine (15 mg/kg) without optic stimulation and rats treated with cocaine with optic stimulation displayed significantly lower values compared with the saline control group (*p* < 0.05), as determined by a two-way ANOVA test [F_OPTIC STIMULATION_ (1,10) = 2.957, *p* > 0.05, F_GROUP_ (1,10) = 28.78, *p* < 0.001, F_INTERACTION_ (1,10) = 0.7083, *p* > 0.05, followed by Tukey’s multiple comparison test]. (*n* = 3 for the saline groups, and *n* = 4 for the cocaine groups). **(B)** The bar histograms depict the level of pERK (Thr202/Tyr204) divided by ERK antibodies; no significant difference was found, as determined by a two-way ANOVA test [F_OPTIC STIMULATION_ (1,10) = 3.882, *p* > 0.05, F_GROUP_ (1,10) = 0.6174, *p* > 0.05, F_INTERACTION_ (1,10) = 2.206, *p* > 0.05, followed by Tukey’s multiple comparison test]. (*n* = 3 for the saline groups, and *n* = 4 for the cocaine groups). **(C)** The bar histograms depict the level of GluA1 divided by Actin, and rats treated with cocaine (15 mg/kg) without optic stimulation and rats treated with cocaine with optic stimulation displayed significantly lower values compared with the saline control group (*p* < 0.05), as determined by a two-way ANOVA test [F_OPTIC STIMULATION_ (1,10) = 5.238, *p* < 0.05, F_GROUP_ (1,10) = 28.43, *p* < 0.001, F_INTERACTION_ (1,10) = 0.3272, *p* > 0.05, followed by Tukey’s multiple comparison test]. (*n* = 3 for the saline groups, and *n* = 4 for the cocaine groups). **(D)** The bar histograms depict the level of GluA1ps845 divided by GluA1 antibodies, and rats treated with cocaine (15 mg/kg) without optic stimulation displayed significantly lower values compared with all other groups (*p* < 0.01), as determined by a two-way ANOVA test [F_OPTIC STIMULATION_ (1,10) = 8.037, *p* < 0.05, F_GROUP_ (1,10) = 11.26, *p* < 0.01, F_INTERACTION_ (1,10) = 12.59, *p* < 0.01, followed by Tukey’s multiple comparison test]. (*n* = 3 for the saline groups, and *n* = 4 for the cocaine groups). **(E)** The bar histograms depict the level of GluA1ps831 divided by GluA1 antibodies, and rats treated with cocaine (15 mg/kg) without optic stimulation displayed significantly lower values compared with the saline control group (*p* < 0.01), and a similar effect was shown in the saline control group with optic stimulation and cocaine treated groups (*p* < 0.01), as determined by a two-way ANOVA test [F_OPTIC STIMULATION_ (1,10) = 6.92, *p* < 0.05, F_GROUP_ (1,10) = 15.83, *p* < 0.01, F_INTERACTION_ (1,10) = 20.12, *p* < 0.01, followed by Tukey’s multiple comparison test]. (*n* = 3 for the saline groups, and *n* = 4 for the cocaine groups). (− O.S refers to - optic stimulation, whereas + O.S refers to + optic stimulation).

In the mPFC, rats treated with cocaine with or without optic stimulation displayed significantly lower values of ERK compared with the saline control group (*p* < 0.05), as determined by a two-way ANOVA test [F_OPTIC STIMULATION_ (1,10) = 2.957, *p* > 0.05, F_GROUP_ (1,10) = 28.78, *p* < 0.001, F_INTERACTION_ (1,10) = 0.7083, *p* > 0.05], followed by Tukey’s multiple comparison test (Figure 4A), while no change in phosphorylated ERK was found (Figure 4B). Additionally, these rats displayed significantly lower values of GluA1 compared with the saline control group (*p* < 0.05), as determined by a two-way ANOVA test [F_OPTIC STIMULATION_ (1,10) = 5.238, *p* < 0.05, F_GROUP_ (1,10) = 28.43, *p* < 0.001, F_INTERACTION_ (1,10) = 0.3272, *p* > 0.05], followed by Tukey’s multiple comparison test (Figure 4C). Furthermore, the rats exhibited significantly lower levels of GluA1ps831 compared with the saline control group (*p* < 0.01), and a similar effect was shown in rats in the saline control group with optic stimulation (*p* < 0.01), as determined by a two-way ANOVA test [F_OPTIC STIMULATION_ (1,10) = 6.92, *p* < 0.05, F_GROUP_ (1,10) = 15.83, *p* < 0.01, F_INTERACTION_ (1,10) = 20.12, *p* < 0.01], followed by Tukey’s multiple comparison test (Figure 4E). Interestingly, enhancing GABAergic tone from the NAc during cocaine conditioning attenuated changes in GluA1 phosphorylation at Ser845 in the mPFC compared with the cocaine treated group (Figure 4D). Rats treated with cocaine without optic stimulation displayed significantly lower values of GluA1ps845 compared with all other groups (*p* < 0.01), as determined by a two-way ANOVA test [F_OPTIC STIMULATION_ (1,10) = 8.037, *p* < 0.05, F_GROUP_ (1,10) = 11.26, *p* < 0.01, F_INTERACTION_ (1,10) = 12.59, *p* < 0.01], followed by Tukey’s multiple comparison test.

Overall, our findings indicate that the modulation of inhibitory inputs from the NAc to the VTA has a significant impact on molecular signaling and glutamatergic neurotransmission in response to cocaine exposure. However, it should be noted that studying the neuroadaptations in rats injected in the NAc presents challenges due to the intricate connections between the NAc and the VTA. Moreover, the injection of AAV-ChR2 in the NAc region, which is crucial for the expression of rewarding effects of drugs such as CPP, may potentially have adverse effects on the vitality and functionality of NAc neurons.

## Discussion

In this study, we utilized optogenetics to specifically stimulate the NAc-VTA GABAergic inputs during the first 15 min of cocaine exposure. As a result, our aim was to investigate the effects of improving GABAergic signaling from the NAc to the VTA during the initial phase of cocaine exposure. The NAc plays a major role in reward processing and motivation in response to both natural rewards and drugs of abuse (Feltenstein and See, 2008). It is well accepted that D1R-expressing and D2R-expressing MSNs play a differential role in promoting reward- and aversion-related behaviors. For example, optical activation of D1R-expressing MSNs has been shown to promote a reward, whereas activation of D2R-expressing MSNs produces aversion (Lobo et al., 2010; Kravitz et al., 2012). Other studies claim that this opposing activity depends on their stimulation pattern, as both D1R- and D2R-expressing MSNs can promote either a reward or an aversion (Soares-Cunha et al., 2020). NAc → VTA inputs synapses onto both GABA and DA neurons but activates different receptors. NAc → VTA projections inhibit GABA neurons via GABA_A_Rs and inhibit DA neurons via GABA_B_Rs. NAc inputs more strongly activate GABA_B_Rs in VTA DA neurons than in GABA neurons, potentially as a result of the differential sensitivity of GABA_B_Rs in GABA and DA neurons (Margolis et al., 2012). In addition, D1R-expressing MSNs were shown to strongly activate GABA_B_-mediated currents in VTA DA neurons (Edwards et al., 2017). This GABA_B_-mediated inhibition was suggested to overcome GABA_A_-mediated inhibition of VTA GABA neurons, which results in the inhibition of VTA DA neuron activity. Studies have shown that pharmacological activation of GABA_B_Rs, using drugs such as baclofen, can reduce the activity of VTA DA neurons. This modulation of GABA_B_Rs has been associated with a reduction in cocaine craving in human (Ling et al., 1998). Additionally, in animal models, activation of GABA_B_Rs has been shown to attenuate the rewarding effects of cocaine (Slattery et al., 2005).

The existence of two modes of inhibitory transmission suggests that disinhibition of DA neurons via GABA_A_Rs and direct inhibition via GABA_B_Rs could “compete” with one another. Considering the perisomatic innervation of NAc → VTA inputs onto DA neurons, we speculate that direct inhibition would override disinhibition. Indeed, we found that activation of NAc → VTA afferent during cocaine exposure resulted in a significant decrease in the expression of cocaine-CPP. This behavioral phenomenon could result from the preferred activation of GABA_B_R in VTA DA neurons. Thus, NAc → VTA inputs may act as a feedback mechanism to prevent overactivation of DA neurons.

### The effect of enhanced the GABAergic tone from the NAc to the VTA during conditioning on cocaine-induced neuroadaptations in reward-related regions

Stimulating the NAc-VTA inputs during cocaine conditioning resulted in a significant decrease in the rats’ preference for the cocaine-associated chamber. This decrease implies that activating NAc afferents interferes with the rewarding effects of cocaine. This interference may be attributed to a reduction in cocaine-induced alterations within the brain’s reward circuitry, extending beyond the VTA to regions such as the NAc and the mPFC.

Our results revealed several significant findings: First, we observed a significant reduction in ERK levels in the VTA of both cocaine groups compared with the saline control group. This suggests that cocaine exposure leads to alterations in ERK signaling in the VTA. Furthermore, optical stimulation of NAc-VTA inputs decreased GluA1 levels in the VTA for animals treated with both saline and cocaine. This reduction can be explained by the activation of GABA_B_R in the dopaminergic cells in the VTA, which leads to reduced activity in VTA DA neurons (Ling et al., 1998) and LTD induction. This is associated with a decrease in cell surface AMPA receptors (Gutlerner et al., 2002), including a reduction in GluA1 levels. These findings indicate that enhancing GABAergic tone from the NAc to the VTA modulates GluA1 expression in this brain region, regardless of cocaine exposure.

In the NAc, optical stimulation of NAc-VTA inputs had different effects depending on cocaine treatment. It significantly increased the ERK levels and decreased GluA1 phosphorylation at Ser845 in the cocaine treated group compared with all other groups. This finding suggests that activating inhibitory inputs from the NAc to the VTA during cocaine conditioning leads to distinct molecular changes in the NAc. Additionally, we observed decreased levels of GluA1 phosphorylation at Ser831 in both cocaine groups compared with the saline control group, indicating that cocaine exposure affects GluA1 phosphorylation at Ser831 in the NAc. The reduction in GluA1 phosphorylation at Ser845 in the cocaine treated group can be explained by the decrease in DA secretion from the VTA, resulting in reduced activation of D1R in the NAc, leading to diminished activation of protein kinase A (PKA), a downstream signaling molecule. Consequently, the reduced PKA activation leads to reduced phosphorylation of GluA1 at S845 (GluA1ps845) (Song and Huganir, 2002). On the other hand, the decrease in GluA1 phosphorylation at Ser831 is likely caused by decreased glutamate secretion from PFC to NAc. This leads to less activation of CaMKII and, subsequently, less phosphorylation of the GluA1 subunit of AMPA receptors at Ser831 (Song and Huganir, 2002).

In the mPFC, exposure to cocaine significantly reduced ERK levels and the levels of GluA1, GluA1ps845, and GluA1ps831. These findings suggest that cocaine exposure has a broad impact on molecular signaling and glutamatergic neurotransmission in the mPFC. Interestingly, enhancing the GABAergic tone from the NAc during cocaine conditioning attenuated changes in GluA1 phosphorylation at Ser845 in the mPFC in the cocaine treated group compared with the cocaine control group. Similarly, enhancing GABAergic tone from the NAc during saline conditioning reduced changes in GluA1 phosphorylation at Ser831 in the mPFC in the saline control group with optic stimulation compared with the saline control group. Enhancing GABAergic tone from the NAc during cocaine conditioning reduced GluA1 levels in the cocaine groups compared with both saline groups, this reduction can be explained by the decrease in DA secretion from dopaminergic cells in the VTA, which leads to reduced D1R activation and LTD induction, and this reduction is associated with a decrease in cell surface AMPA receptors (Gutlerner et al., 2002), including a reduction in GluA1 levels.

It is important to acknowledge that studying neuroadaptations in rats injected in the NAc presents challenges due to the interconnected nature of this brain region. For example, changes in the VTA can influence the NAc and vice versa, making it difficult to attribute specific effects solely to the NAc. Additionally, other brain regions, such as the PFC, also play important roles in reward processing and can interact with the NAc and the VTA.

Overall, our study unveiled that augmenting the inhibitory inputs from the NAc to the VTA exerts a significant influence on the rewarding properties of cocaine, and the molecular signaling and glutamatergic neurotransmission are involved in cocaine exposure.

## Data availability statement

The raw data supporting the conclusions of this article will be made available by the authors, without undue reservation.

## Ethics statement

The animal study was approved by the IACUC of the Hebrew University. The study was conducted in accordance with the local legislation and institutional requirements.

## Author contributions

AK: Conceptualization, Data curation, Investigation, Methodology, Project administration, Resources, Software, Supervision, Validation, Writing – original draft. RY: Conceptualization, Data curation, Investigation, Methodology, Project administration, Resources, Software, Supervision, Validation, Writing – original draft.
